# Supplementary material for: 5-Bromo-3,4-dihydroxybenzaldehyde Promotes Hair Growth through Activation of Wnt/β-Catenin and Autophagy Pathways and Inhibition of TGF-β Pathways in Dermal Papilla Cells
Source: Molecules. 2022 Mar 28;27(7):2176. doi: 10.3390/molecules27072176 (PMC9000556; doi:10.3390/molecules27072176)
Supplement: Supplementary file 1 [file molecules-27-02176-s001.zip › molecules-1631790-supplementary.pdf]

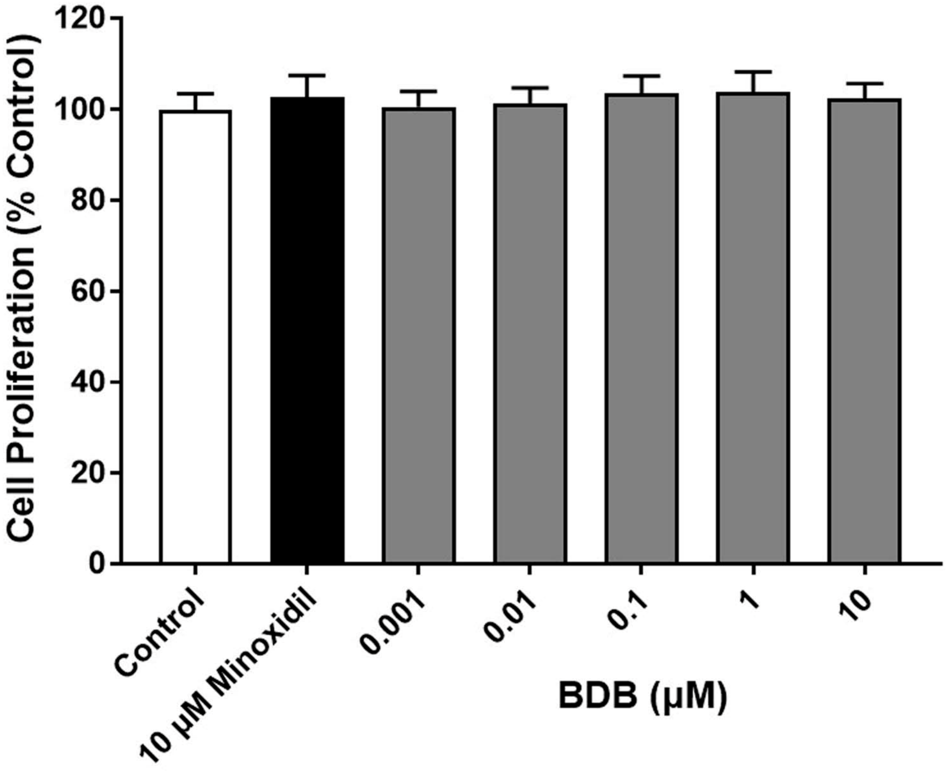

Supplementary Figure S1. Effects of BDB on the proliferation of DPCs. DPC proliferation stimulated using various concentrations of BDB or minoxidil for 24 h. Data are presented as mean ± SD.

Supplementary Table S1. List of antibodies used for immunoblotting.

Antibodies	Supplier	Species	Dilution
phospho-(ser552)-β-catenin	Cell Signaling	Rabbit	1:1000
phospho-(ser675)-β-catenin	Cell Signaling	Rabbit	1:1000
β-catenin	Santa Cruz	Rabbit	1:2000
phospho-(ser9)-GSK3β	Cell Signaling	Rabbit	1:1000
Cyclin E	Santa Cruz	Rabbit	1:1000
Cyclin D1	BD Biosciences	Mouse	1:1000
Phospho-(Thr160)-CDK2	Cell Signaling	Rabbit	1:1000
CDK2	Santa Cruz	Rabbit	1:1000
Smad2/3	Santa Cruz	Mouse	1:1000
Phospho-Smad2	Cell Signaling	Rabbit	1:1000
Phospho-Smad3	Cell Signaling	Rabbit	1:1000
Atg7	Cell Signaling	Rabbit	1:1000
LC3A/B	Cell Signaling	Rabbit	1:1000
Atg5	Cell Signaling	Rabbit	1:1000
Atg16L	Cell Signaling	Rabbit	1:1000
Lamin B1	Abcam	Rabbit	1:2000
β-Actin	Sigma-Aldrich	Mouse	1:5000
